# Association of *MICA*-129Met/Val polymorphism with clinical outcome of anti-TNF therapy and MICA serum levels in patients with rheumatoid arthritis

**DOI:** 10.1038/s41397-020-0164-3

**Published:** 2020-03-03

**Authors:** Milena Iwaszko, Jerzy Świerkot, Marta Dratwa, Barbara Wysoczańska, Lucyna Korman, Bartosz Bugaj, Katarzyna Kolossa, Sławomir Jeka, Piotr Wiland, Katarzyna Bogunia-Kubik

**Affiliations:** 1 Laboratory of Clinical Immunogenetics and Pharmacogenetics, Hirszfeld Institute of Immunology and Experimental Therapy, Polish Academy of Sciences, Wrocław, Poland; 2 Department of Rheumatology and Internal Medicine, Wrocław Medical University, Wrocław, Poland; 3 Jan Biziel University Hospital No. 2, Department of Rheumatology and Connective Tissue Diseases, Bydgoszcz, Collegium Medicum in Bydgoszcz, UMK, Torun, Poland

**Keywords:** Genetic markers, Autoimmune diseases, Rheumatic diseases, Genetic association study

## Abstract

MHC class I polypeptide-related sequence A (MICA) is a stress-induced protein involved in activation of NK and T cells through interaction with NKG2D receptor. These molecules are atypically expressed in synovium of patients diagnosed with rheumatoid arthritis (RA). A total of 279 patients with RA, qualified to TNF-blockade therapy, were genotyped for *MICA* rs1051792 SNP. The effectiveness of anti-TNF agents was assessed with European League Against Rheumatism criteria. Significant relationship between *MICA* rs1051792 and outcome of TNF-blockade therapy has been found. The *MICA* rs1051792 *GG* genotype was overrepresented in patients non-responsive to anti-TNF drugs in comparison with other genotypes (*p* = 0.010). On the other hand, beneficial therapeutic response was more frequently detected among RA subjects possessing heterozygous genotype than those with homozygous genotypes (*p* = 0.003). Furthermore, increased MICA concentrations in serum were observed in patients possessing *MICA* rs1051792 *GG* genotype as compared with those with *GA* or *AA* genotypes (*p* = 1.8 × 10^−5^). The results from this study indicate the potential influence of *MICA* rs1051792 polymorphism on modulation of therapeutic response to TNF-blockade treatment in RA.

## Introduction

Rheumatoid arthritis (RA) is a chronic inflammatory disorder with a worldwide prevalence of around 1% and predominance in females [1, 2]. The hallmark of this autoimmune disease is persistent inflammation of the synovium resulting in cartilage and bone destruction. RA belongs to systemic conditions, and might also affect other tissues and organs. Extra-articular manifestations encompass cardiovascular and pulmonary disorders, rheumatoid nodules, ophthalmologic manifestations, vasculitis, neuropathy and amyloidosis [3]. Disease progression leads ultimately to functional disability, premature mortality as well as economic and social burdens [4, 5]. Although the RA aetiology is multifactorial and remains elusive to date, it has been established that both genetic and environmental components are involved in RA development [1]. It is estimated that genetic background accounts for ~50% of the risk of developing RA [6, 7].

In the field of RA treatment, significant progress has been achieved with the introduction of anti-TNF biologic therapy [8]. Although TNF-blockade therapy constitutes a spectacular advance in the RA treatment, approximately one-third of patients do not respond to this therapeutic approach [9, 10]. Biologic mechanisms underlying this non-responsiveness to anti-TNF treatment remain obscure, although inefficiency of therapy might be partially determined by genetic heterogeneity among patients. Polymorphic variants engaged in modulation of outcome of the treatment with TNF inhibitors may contribute to prediction of anti-TNF response. Due to considerable heterogeneity in response rates to these agents among patients, identification of predictive biomarkers is critical to optimize application of anti-TNF therapy. Predictors of anti-TNF treatment outcome could contribute to selection of patients before initiation of therapy, leading to improvement of the effectiveness of anti-TNF agents and reduction of costs, as well as considerable adverse effects related with therapy [11, 12].

The MICA gene is situated within human leucocyte antigen (HLA) region that also encompasses HLA-DRB1 locus, regarded as a strong genetic factor affecting predisposition to the development of RA [13, 14]. MICA represents a most polymorphic HLA class IB locus [15]. Protein encoded by MICA gene structurally resembles classical MHC class I molecules, although it does not associate with β2-microglobulin, has an extremely narrow peptide-binding groove and in consequence no role in peptide presentation [16]. It functions as a stress-inducible molecule and affects a response of innate as well as acquired immune system. Constitutive tissue distribution of MICA protein is limited to fibroblasts, intestinal epithelium as well as endothelial cells [17]. However, MICA expression is triggered under pathologic circumstances comprising cellular stress, tumorigenesis or pathogen infection [18–20].

MICA constitutes a ligand for natural killer (NK) cell receptor NKG2D, expressed on NK as well as T lymphocytes [20, 21]. Activatory signal transduced by MICA-NKG2D interaction stimulates NK cells and provides co-stimulatory signal to T-cell subsets [22, 23]. The MICA molecule constitutes a key component of a microbial and tumour surveillance [24]. Engagement of NKG2D by MICA unconditionally triggers transduction of the activating signal, leading to elimination of dysfunctional cells [20, 25]. This signalling pathway constitutes a crucial defence mechanism involved in detection and eradication of infected, tumorous or stress-induced cells. The presence of MICA ligands on target cells stimulates NK and T-cell responses comprising cellular cytotoxicity, inflammatory cytokine production as well as cellular proliferation [21, 26–28]. Nevertheless, the MICA-NKG2D pathway dysregulation might lead to aberrant activation of effector cells and promote autoimmune pathology [25, 29].

Aberrant expression of MICA molecules has been detected on RA synoviocytes [30]. Also, sera of RA patients contain a considerable amount of synoviocyte-derived soluble MICAs [29]. In addition, clonal expansion of unique subpopulation of CD4+ T lymphocytes negative for CD28 receptor is detected in peripheral blood and synovial tissue of RA patients [31]. Substantial percentage of CD4+CD28− T cells expresses the NKG2D molecule; however, this receptor is absent on conventional CD4+ T cells [30]. Interaction between NKG2D presented on these cells and MIC ligands aberrantly expressed on RA synovial tissue, may promote autoimmune response towards RA synoviocytes.

The significance of *MICA* polymorphisms has been linked to a development of autoimmune conditions, cancer as well as pathogen infections [18, 32–34]. The *MICA* rs1051792 constitutes a genetic variation of documented functional effect. This non-synonymous polymorphism is located in exon 3, and comprises single base change (G/A), resulting in replacement at amino-acid position 129 in the alpha 2-heavy-chain domain of MICA protein, where a methionine (Met) substitutes a valine (Val). It has been reported that *MICA* rs1051792 affects the affinity of binding MICA molecules to the NKG2D receptor and NKG2D signalling [35, 36]. The role of this SNP has been investigated in studies regarding malignant diseases [35, 37, 38], pathogen infections [38–40], recurrent miscarriage [41] as well as autoimmune disorders, including RA [42–44], ankylosing spondylitis (AS) [45], type I diabetes [46], inflammatory bowel disease (IBD) [47, 48], systemic lupus erythematosus [44] and psoriasis [49]. However, in accordance to our knowledge, the significance of *MICA* polymorphism with respect to the effectiveness of anti-TNF biological drugs in the treatment of RA has not been investigated to date.

The purpose of the study was to investigate a potential association between the *MICA* rs1051792 genetic variants and response to treatment with TNF inhibitors. Possible relationships of MICA serum levels among patients with respect to *MICA* polymorphism were also investigated.

## Materials and methods

### Patients

Two hundred and seventy-nine unrelated patients diagnosed with RA were enroled to the study. Of these, ten individuals were lost to follow-up at the 6th month of the therapy, and consequently excluded from the respective analyses. Each patient satisfied the 1987 American College of Rheumatology criteria [50], and was qualified to anti-TNF therapy. The inclusion criteria for participation in the study comprised high-disease activity (Disease Activity Score in 28 joints (DAS28) ≥5.1) before initiation of TNF-blockade treatment, failure to respond to at least two disease-modifying antirheumatic drugs (DMARDS), age over 18, Caucasian origin and complete medical records. Applied exclusion criteria were as follows: kidney or liver insufficiency, concurrent autoimmune disorders, drug-resistant infectious disease, cancer diagnosis, diabetes, alcohol or drug addiction, pregnancy, incomplete medical data and reluctance to cooperation.

Clinical evaluation of patients comprised values of C-reactive protein (CRP) and erythrocyte sedimentation rate (ESR), number of swollen and tender joints, anti-cyclic citrullinated peptide antibodies (anti-CCP) and rheumatoid factor (RF) levels, measurement of disease activity (DAS28), measurement of pain intensity (visual analogue scale (VAS), range: 0–100 mm) and global health evaluation provided by a physician as well as a patient.

The anti-TNF agents were administered to the patients following standard protocols: 3 mg/kg of body weight of infliximab intravenously at weeks 0, 2 and 6, then every 2 months thereafter; 40 mg of adalimumab subcutaneously every other week; 50 mg of etanercept subcutaneously every week; 400 mg of certolizumab pegol subcutaneously at weeks 0, 2 and 4, then 200 mg every second week thereafter.

Most of the patients were comedicated with non-steroidal anti-inflammatory drugs, glucocorticoids and DMARDs. Amongst the patients, 91% were treated with glucocorticoid prednisone (mean dose: 6.6 mg/day) and 92% received methotrexate (mean dose: 22 mg/week).

RA activity was assessed using a validated, combined index comprising four components, including tender joint count, swollen joint count, global health assessment by the patient (VAS, mm) and the result of the laboratory test: CRP or ESR level. In accordance to a value of the DAS28 score, the patients were stratified into three subgroups: those with DAS28 > 5.1 were considered to have high-disease activity; a moderate disease activity was assumed for patients in the range of 3.2 < DAS28 ≤ 5.1; those with DAS28 ≤ 3.2 were considered to possess low-disease activity.

The European League Against Rheumatism (EULAR) criteria were used to assess the clinical outcome of anti-TNF treatment after 3 as well as 6 months. These criteria are calculated according to a combination of DAS28 change between baseline and time of assessment with respect to DAS28 value reached at the time of evaluation [51]. Calculations of EULAR response were performed after 3 as well as 6 months after a commencement of the treatment with TNF inhibitors. A response was considered as good when reduction of a DAS28 score value (ΔDAS28) of more than 1.2 was accompanied by a post-treatment value of the DAS28 lower or equal to 3.2. A response was interpreted as intermediate in two cases: when a ΔDAS28 value was more than 1.2 and a post-treatment value of the DAS28 was higher than 3.2, or when a value of the ΔDAS28 was between 0.6 and 1.2, and a value of the DAS28 at the time of assessment was lower or equal to 5.1. Finally, a lack of response was assumed when a ΔDAS28 value was lower than 0.6, as well as when a ΔDAS28 value lower than 1.2 was accompanied with a post-treatment value of the DAS28 higher than 5.1.

The study obtained approval from the local research ethical committee (Wrocław Medical University Ethics Committee). All participants of the study provided written informed consents.

### *MICA* rs1051792 genotyping

Whole-blood samples were collected in ethylenediaminetetraacetic acid anticoagulant tubes. Genomic DNA was obtained with Maxwell 16 Blood DNA Purification Kit (Promega Corp., Madison, WI, USA) according to the manufacturer’s instructions. Discrimination of *MICA* genetic variants was performed on LightCycler 480 II instrument (Roche Applied Science, Mannheim, Germany) using single-nucleotide identification polymorphism (SNiP) reagent (LightSNiP) designed and provided by TIB-MolBiol (TIB-MolBiol, Berlin, Germany). LightSNiP assay is based on real-time PCR and probe-melting analysis. Genotyping was performed following the recommendations of the manufacturer.

### Determination of MICA levels in serum of RA patients

Serum samples from patients diagnosed with RA were gathered prior to the initiation of anti-TNF therapy. A total of 54 serum samples were obtained and used in the analysis of MICA concentrations. Serum MICA concentrations were detected employing Human Magnetic Luminex Assay (R&D Systems Inc., Minneapolis, MN, USA) using Luminex 200 system (Luminex Corp., Austin, TX, USA). All measurements were performed following the guidelines of the manufacturer.

### Statistical analysis

Demographic data and baseline clinical parameters of RA patients were represented as mean and standard deviation for quantitative data, while categorical data were described using frequencies and proportions. Genotype frequencies of the MICA genetic variant in patients’ group were tested for conformance with the Hardy–Weinberg model employing population genetics package for R (cran:genetics, version 1.3.8.1). Response to TNF-blockade therapy among patients was estimated in accordance with EULAR response criteria. Evaluation of anti-TNF treatment efficacy was performed after 3 as well as 6 months following initiation of the therapy. The patients were ascribed to one of the following groups: with good response to therapy, with intermediate response or lack of response. Distribution of the alleles and genotypes of MICA genetic variant with respect to the response to TNF inhibitors achieved by the patients were assessed by employing Fisher’s exact test. Relationships between MICA allele and genotype frequencies, and selected baseline clinical data, were analyzed with parametric Fisher’s exact test for categorical data or non-parametric Mann–Whitney test for continuous variables. To assess the influence of MICA polymorphism on MICA concentrations in serum, Mann–Whitney test was also employed. All applied tests were two-tailed, and statistical significance cutoff of 0.05 was assumed. We estimated that for variants with minor allele frequency 0.3, our sample size allows us to achieve >80% power to detect the effects >2.0. All statistical analyses were conducted using R software environment (version 3.3.1; x86_64-pc-linux-gnu) [52], except power calculation where Quanto software (v1.2.4, http://biostats.usc.edu/Quanto.html) was employed.

## Results

### Baseline characteristics of studied subjects and outcome of TNF-blockade treatment

Baseline demographic as well as clinical data of studied subjects are presented in Table 1. Two hundred and seventy-nine patients with RA qualified to biologic therapy were included in the study. Females constituted 78.5% of the group. There were 65.9% patients seropositive for RF, and 95.4% of them were anti-CCP positive. The studied patients had mean (±SD) age of 51.6 (±12.3) years at the time of enrolment to the study. The mean (±SD) duration of RA equalled to 12.6 (±8.1) years, and the onset of the disease was at the age of 39.2 (±12.0) years.

**Table 1:** Description of patients’ cohort.

Patients with RA	*N* = 279
Demographics and clinical data	
Mean age, years (±SD)	51.6 (±12.3)
Females (%)	78.5
Smokers (%)	33.3
Mean age of rheumatoid arthritis onset, years (±SD)	39.2 (±12.0)
Mean duration of rheumatoid arthritis, years (±SD)	12.6 (±8.1)
Mean Disease Activity Score in 28 joints (±SD)	6.5 (±0.6)
Mean C-reactive protein level, mg/l (±SD)	24.4 (±35.7)
Anti-citrullinated peptide antibodies positive (%)	95.4
Rheumatoid factor positive (%)	65.9
Therapeutic drugs	
Anti-TNF agents	
Etanercept (%)	54
Adalimumab (%)	33
Infliximab (%)	7
Certolizumab pegol (%)	6
Methotrexate (%)	92
Steroids (%)	91

Overall, good response to treatment with TNF inhibitors after 3 months was observed in 10.0% patients, moderate response in 81.7% patients, and 8.3% of patients were unresponsive to therapy. After 6 months of therapy, 48.3% of patients were classified as good responders, 5.2% as non-responders, while intermediate response was noticed in 46.5% of patients.

### Relationships between *MICA* rs1051792 genetic variant and clinical outcome of anti-TNF agents after 3 and 6 months

The clinical outcome of treatment with anti-TNF agents with respect to the *MICA* rs1051792 polymorphism is given in Table 2. A statistically significant relationship between *MICA* rs1051792 genetic variants, and efficacy of treatment with TNF inhibitors, has been found after 3 months. Failure of the TNF-blockade therapy was associated with the presence of the homozygous *GG* (Val/Val) genotype. This *MICA GG* (Val/Val) homozygosity was significantly overrepresented in patients non-responsive to TNF-blockade therapy in comparison with patients possessing the *AA* (Met/Met) or *GA* (Val/Met) genotypes (*p* = 0.014, OR = 3.06, CI_95%_ = [1.17, 8.66]). Furthermore, a significant association of the *MICA* rs1051792 heterozygous genotype, and clinical outcome after 3 months, has been detected. Patients bearing the *MICA* rs1051792 heterozygous genotype displayed beneficial responses to the treatment with TNF inhibitors as compared with patients possessing homozygous *AA* (Met/Met) or *GG* (Val/Val) genotypes (*p* = 0.004, OR = 4.65, CI_95%_ = [1.49, 19.33]).

**Table 2:** Clinical outcome of TNF-blockade treatment after 3 and 6 months in relation to genotype and allele frequencies of the *MICA* rs1051792.

	Response to anti-TNF therapy			
*MICA* rs1051792	After 3 months		After 6 months	
	No response [number (%)]	Good/moderate response [number (%)]	No response [number (%)]	Good/moderate response [number (%)]
G	34 (73.9%)	321 (62.7%)	16 (57.1%)	323 (63.3%)
A	12 (26.1%)	191 (37.3%)	12 (42.9%)	187 (36.7%)
GG	15 (65.2%)^a^	97 (37.9%)^a^	4 (28.6%)	101 (39.6%)
GA	4 (17.4%)^b^	127 (49.6%)^b^	8 (57.1%)	121 (47.5%)
AA	4 (17.4%)	32 (12.5%)	2 (14.3%)	33 (12.9%)

*OR* odds ratio, *95% CI* 95% confidence interval.

^a^*GG* vs *AA* + *GA*, *p* = 0.014, OR = 3.06, 95% CI (1.17, 8.66).

^b^*GA* vs *AA* + *GG*, *p* = 0.004, OR = 0.21, 95% CI (0.05, 0.67).

The analysis of the distribution of the *MICA* rs1051792 alleles after 6 months of implementation of anti-TNF therapy did not reveal any significant differences with respect to efficacy of treatment. Also, no statistically significant correlation between genotype frequencies of the *MICA* rs1051792 polymorphism and clinical outcome of treatment with anti-TNF medications at the 6th month has been detected.

### Relationships between *MICA* rs1051792 gene polymorphism and baseline parameters of disease activity of studied subjects

Analyses of possible associations of the *MICA* rs1051792 genetic variant and baseline clinical features, including DAS28, CRP, RF and anti-CCP, were also performed (Table 3). The frequencies of the *MICA* rs1051792 alleles and genotypes did not differ among patients with respect to the presence of anti-CCP antibodies. There were also no significant differences between patients seropositive and seronegative for RF with regard to *MICA* rs1051792 alleles and genotypes. Moreover, analyses of baseline DAS28 scores and distribution of alleles and genotypes of *MICA* rs1051792 polymorphism did not reveal any association. In addition, no significant relationship between baseline CRP values and *MICA* rs1051792 polymorphism was exposed.

**Table 3:** Genotype and allele frequencies of the *MICA* rs1051792 among RA patients with respect to baseline clinical data.

*MICA* rs1051792	DAS28 at baseline [mean (±SD)]	CRP at baseline [mean (±SD)]	RF+ [number (%)]	CCP+ [number (%)]
G			227 (66.6%)	300 (96.2%)
A			131 (65.8%)	166 (94.3%)
GG	6.6 (±0.61)	24.6 (±39.02)	72 (67.9%)	95 (96.0%)
GA	6.5 (±0.62)	25.2 (±35.85)	83 (64.3%)	110 (96.5%)
AA	6.5 (±0.70)	20.1 (±19.17)	24 (68.6%)	28 (90.3%)

*DAS28* disease activity score 28, *CRP* C-reactive protein; *RF* rheumatoid factor, *anti-CCP* anti-cyclic citrullinated peptide antibodies.

### Relationships between *MICA* rs1051792 gene polymorphism and MICA serum expression levels in studied subjects

**Fig. 1:**
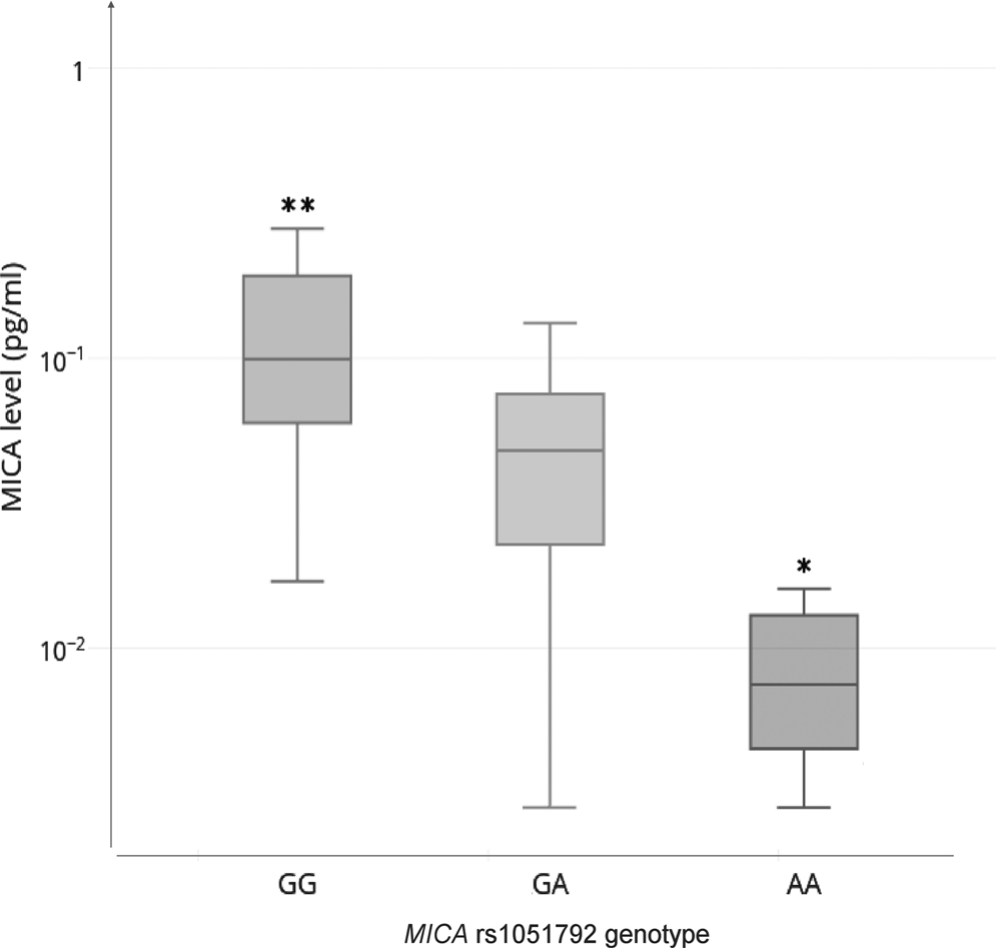
Distribution of serum MICA levels with respect to the *MICA* rs1051792 genotypes. *AA vs *GA* + *GG*, *p* = 3.8 × 10^−6^, *W* = 59.5; **GG vs *AA* + *GA*, *p* = 1.8 × 10^−5^, *W* = 569.5; *Y* axis in logarithmic scale.

Relationships between the distribution of genotypes and alleles of *MICA* rs1051792 polymorphism and MICA serum expression levels were also determined. Fifty-four patients were examined with respect to MICA serum levels. Significant differences in serum expression levels between *MICA* rs1051792 genotypes were observed (Fig. 1). Increased MICA concentrations in serum were detected among patients possessing the *MICA* rs1051792 *GG* (Val/Val) genotype than in those with the *GA* (Val/Met) or *AA* (Met/Met) genotypes (*p* = 1.8 × 10^−5^, *W* = 569.5). Moreover, the presence of the *MICA* rs1051792 *AA* (Met/Met) genotype among patients correlated with significantly lower MICA serum levels as compared with the other genotypes (*p* = 3.8 × 10^−6^, *W* = 59.5).

## Discussion

The MICA molecule has been established as a significant component of both innate and acquired immunity. It is involved in activation of NK and T cells through interaction with NKG2D receptor. Since MICA is expressed under pathological conditions, it constitutes a significant part of host defence system, resulting in detection and eradication of abnormal cells. Dysregulation of the signalling pathway mediated by MICA molecules may trigger self-aggression and promote proinflammatory process underlying the development of autoimmune diseases.

The MICA molecules are atypically expressed on RA synovium, and are present in abundance in sera of RA patients [29, 30]. Although MICA proteins act as indicators of dysfunctional cells, prolonged MICA expression leads to decreased NKG2D levels and impairment of MICA–NKG2D interaction to avoid exacerbate NK and T cells stimulation and autoimmune reactions [53, 54]. However, it has been revealed that in the case of RA, chronic exposure to MIC levels does not induce the downregulation of NKG2D expression, implying that such self-regulatory mechanism is not present in RA [30]. It was hypothesized that ligand-induced downmodulation of NKG2D expression may be overcome by the opposing effect of TNF-α and IL-15 that are copious in inflamed synovium and constantly upregulate NKG2D expression [30, 55].

Moreover, patients with RA are characterized by the presence of an unusual subpopulation of CD4+ T lymphocytes negative for CD28 receptor in peripheral blood and inflamed synovium [31]. These autoreactive cells lost CD28 expression and majority of them acquired expression of the NKG2D receptor [30, 56]. Since anomalous levels of MIC molecules are expressed on RA synoviocytes, these cells may contribute through the MICA–NKG2D-signalling pathway to RA development and progression [30]. The CD4 + CD28 lymphocytes secrete high amounts of cytokines (mainly IFN-γ and TNF-α) and exhibit cytotoxic activity [31, 57]. This subpopulation is also characterized by resistance to apoptosis and tissue infiltration capacity [56, 58]. In addition, a positive correlation between CD4+CD28− T cells’ expansion, and RA severity, has been observed [59, 60]. The presence of the unique subpopulation of CD4+ T lymphocytes has also been reported in other autoimmune conditions, such as multiple sclerosis, AS or IBD [61–63].

In the current research, *MICA* rs1051792 polymorphism was associated with the efficacy of anti-TNF-blockade therapy. Lack of therapeutic effect after 3 months of treatment was more frequently detected among patients bearing the *MICA* rs1051792 *GG* genotype than patients possessing *GA* and *AA* genotypes. On the other hand, the presence of the heterozygous *GA* genotype among patients correlated with better clinical outcome of TNF-blockade therapy after 3 months. In line with these results, the *MICA* rs1051792 polymorphism was associated with predisposition to RA development in a study conducted by Kristen et al. [43]. In this study frequency of the *MICA* rs1051792 *GG* homozygous individuals was higher among RA subjects in comparison with controls. It should be noted that the *MICA* rs1051792 polymorphism was investigated indirectly in this study as *MICA* rs1051794 was examined, which is in complete linkage disequilibrium (LD) with *MICA* rs1051792. In addition, correlation between the presence of RF and *MICA* rs1051792 *GG* homozygosity was observed in RA patients in a study by Achour et al., however, this study did not expose significant association between *MICA* rs1051792 polymorphism and predisposition to RA [42]. Moreover, the deleterious role of *MICA* rs1051792 *GG* genotype was reported in subjects diagnosed with another autoimmune disease—ulcerative colitis (UC). The *MICA* rs1051792 *GG* genotype as well as the *G* allele was prevalent among patients with UC in comparison with healthy individuals [47]. Also, similar results were documented with regard to diabetes mellitus (DM) patients [46]. In the study conducted by Raache et al., a relationship between *MICA* rs1051792 polymorphism and predisposition to DM was detected. Both the *MICA* rs1051792 *G* allele and the *GG* homozygous genotype were more frequently observed in DM patients than in healthy group.

The *MICA* rs1051792 non-synonymous polymorphism is positioned in exon 3 and comprises single base change (G/A) leading to substitution of Met for Val at position 129 of the alpha 2-heavy chain domain of MICA protein. This substitution was implicated in functional differences of MICA protein. It has been revealed that *MICA* rs1051792 polymorphism modulates a strength of MICA binding to NKG2D receptor probably by a conformational change resulted from amino-acid replacement [36]. Proteins encoded by *MICA*-129Met have been characterized to bind NKG2D with higher avidity. On the other hand, decreased binding affinity to NKG2D has been attributed to MICA-129 isoform containing valine. It has been documented that MICA rs1051792 isoforms also affect NKG2D signalling, and they display different capacity to induce NKG2D-mediated effector cells responses [35]. The MICA-129Met variant was associated with increased NKG2D signalling in comparison with MICA-129Val. The presence of MICA protein containing Met resulted in enhanced NK-mediated cytotoxicity and IFN-γ secretion as well as faster costimulation of CD8+ T cells. However, subsequent observations imply that the ultimate functional effect of *MICA* genetic variants on effector cells’ functions is dependent on the amount of MICA ligands on a cell surface. It has been documented that the increase of MICA expression intensity on target cells positively correlated with cytotoxic ability of NK cells and cytokine secretion only in the case of the presence of MICA-129Val variant [35]. Furthermore, augmented amounts of MICA-129Val molecules resulted in increased co-stimulatory activation of CD8+ T cells. These effects were not observed with respect to the MICA-129Met variant. Augmented MICA-129Met expression levels were associated neither with increased NK-cell nor CD8+ T cell functions. Moreover, high-expression intensities of MICA-129Met induced decreased expression of NKG2D receptors both on NK and CD8+ T lymphocytes resulting in disruption of MICA–NKG2D interaction [35]. It seems that such negative feedback signal triggered by MICA-129Met might limit a strong signal transduced by this variant in order to prevent exacerbated NKG2D-mediated immune response. These results suggest that increased NKG2D signalling is mediated by MICA-129Met variant at low-expression levels in comparison with MICA-129Val variant, but contradictory effect is observed when augmented amounts of MICA-129Met molecules are present on cells surface [34]. In view of the finding that high-expression levels of MICA-129Val variants do not act as stimulators of NKG2D receptors’ downregulation, the presence of an augmented amount of this variant on the target cells’ surface might mediate transmission of increased activatory signal via NKG2D receptors to effector cells. It might trigger excessive NK and T cell responses and in consequence contribute to autoimmune reaction leading to RA development.

In the current study a significant relationship between *MICA* rs1051792 polymorphism and serum expression levels in RA patients was also observed. The presence of the *MICA* rs1051792 *G* allele positively correlated with MICA serum levels. Moreover, patients carrying the *MICA* rs1051792 *GG* homozygous genotype exhibited increased serum MICA concentrations as compared with those possessing *GA* and *AA* genotypes. Consistent results were obtained in a study regarding patients with UC. Increased serum MICA levels were more frequently detected among UC patients bearing homozygous *MICA* rs1051792 *GG* genotype than other genotypes [47]. These findings are supported by a study investigating a potential impact of *MICA* rs1051792 polymorphism on MICA cell surface expression. Increased intracellular retention of the MICA-129Met variant in comparison with MICA-129Val has been observed, leading to reduced expression intensity of MICA-129Met variant on a plasma membrane [64].

Taking into account the mentioned disruption of MICA-induced downmodulation of NKG2D expression reported in RA, it may be speculated that NKG2D levels are not reduced in response to the chronic expression of MICA molecules not only due to increased TNF activity, but it may also be associated with the presence of MICA-129Val variant at high-expression levels on target cells. In this context, anti-TNF therapy neutralizes impact of TNF on this mechanism, however, the presence of augmented amounts of MICA-129Val may still dysregulate ligand-induced downmodulation of NKG2D expression, leading to perpetuation of exaggerated levels of this receptor on effector cells. In consequence, patients carrying *MICA*-129Val genotype may achieve worse response to treatment.

The current study is the first to demonstrate a relationship of the *MICA* rs1051792 with the efficacy of TNF-blockade therapy in RA. According to our knowledge, no other studies published to date investigated the potential influence of the *MICA* rs1051792 polymorphism on response to treatment with anti-TNF agents. Only one study, by Martinez et al., investigated trinucleotide repeat polymorphism (GCT)*n* within the transmembrane region of MICA in the context of the response to infliximab therapy among RA patients [65]. However, the present study possesses some limitations that should be considered when regarding the obtained results. It should be noted that other genetic variants, remaining in LD with the investigated SNP, might influence the aforementioned associations. Also, the research was based on a relatively limited number of RA patients involved, and it adopted 0.05 level of statistical significance that might be liberal. Therefore, further studies in larger patient cohorts from other ethnic groups are indispensable to confirm involvement of this polymorphism in determination of therapeutic response to TNF-blockade agents.

In conclusion, the results obtained in the present study contribute to the current state of knowledge on the role of genetic factors in Polish patients diagnosed with RA [66–68], and indicate an involvement of *MICA* rs1051792 polymorphism in the determination of therapeutic response to anti-TNF biological drugs in RA patients. Inefficiency of therapy after 3 months has been significantly associated with presence of the *MICA* rs1051792 *GG* genotype among RA patients receiving anti-TNF agents. Furthermore, the response to TNF-blockade agents was more frequently observed among patients bearing the heterozygous *GA* genotype than patients with homozygous genotypes. Also, the presence of the *MICA* rs1051792 *GG* genotype correlated with increased MICA concentrations in serum of RA patients. The obtained results indicate that the *MICA* rs1051792 genetic variant might constitute potential candidate locus to predict response to TNF-blockade therapy in patients with RA.
